# PGPR–AMF consortia improve drought tolerance in maize through stomatal regulation, antioxidant defense, and yield stability

**DOI:** 10.3389/fpls.2026.1802481

**Published:** 2026-06-23

**Authors:** Oscar Mauricio Chenche-López, Cesar Manuel Chenche-López

**Affiliations:** Universidad Estatal de Milagro, Milagro, Ecuador

**Keywords:** drought tolerance, PGPR–AMF consortia, redox homeostasis, water deficit, *Zea mays*

## Abstract

**Introduction:**

Water deficit is a major abiotic constraint on maize productivity and is expected to intensify under increasing climate variability. This study evaluated whether inoculation with plant growth-promoting rhizobacteria (PGPR), arbuscular mycorrhizal fungi (AMF), or their consortium could mitigate moderate and severe water deficit in *Zea mays*.

**Methods:**

A 4 × 3 factorial completely randomized design was implemented, comprising four inoculation treatments and three water regimes, with three biological replicates per treatment combination (36 experimental units). Physiological, biochemical, growth, and yield-related traits were jointly analyzed using two-way ANOVA, correlation analysis, and principal component analysis (PCA).

**Results:**

The first two PCA dimensions explained 54.0% of the total variance and separated a productivity-related axis, characterized by photosynthesis, chlorophyll status, relative water content, and yield, from an oxidative-stress axis dominated by malondialdehyde and proline. Under severe water deficit, the PGPR–AMF consortium maintained 10.7% higher relative water content and 19.5% higher yield than the non-inoculated control, while sustaining higher photosynthetic activity and lower oxidative damage. Two-way ANOVA revealed significant effects of inoculation treatment and water regime on yield, photosynthesis, relative water content, and SOD activity, whereas proline and MDA were primarily influenced by stress intensity.

**Discussion:**

The results demonstrate that PGPR–AMF inoculation mitigates drought effects in maize through coordinated maintenance of plant water status, gas exchange, antioxidant regulation, and productive stability. These findings support the use of microbial consortia as a sustainable strategy to enhance maize resilience under increasing water-limitation scenarios.

## 1 Introduction

Water deficit is one of the main abiotic constraints limiting maize productivity and is expected to intensify under increasing climatic variability. In maize, reduced water availability disrupts plant water status, restricts stomatal conductance and carbon assimilation, and ultimately reduces biomass accumulation and grain yield. Drought also promotes oxidative imbalance, which is commonly reflected in altered antioxidant activity and membrane damage markers such as SOD, CAT, POD, and MDA (Hasanuzzaman et al., 2020; Mittler et al., 2022).

Plant-associated beneficial microorganisms have emerged as a promising strategy to mitigate these effects. PGPR can improve root growth, nutrient acquisition, and stress signaling, whereas AMF can enhance water and nutrient uptake through the extraradical mycelial network and contribute to antioxidant regulation under drought. Recent evidence in maize indicates that microbial inoculation can preserve gas exchange, relative water content, and antioxidant capacity under water-limited conditions (Notununu et al., 2024).

Current interest has increasingly shifted from single inoculations to microbial consortia, because PGPR–AMF co-inoculation may provide more integrated drought mitigation than either partner alone. In maize grown under drier soil, co-inoculation has been associated with improved tolerance, lower cellular injury, enhanced microbial functioning belowground, and higher yield-related performance than non-inoculated plants (Khan et al., 2024; Zhang et al., 2025).

**Figure 1:**
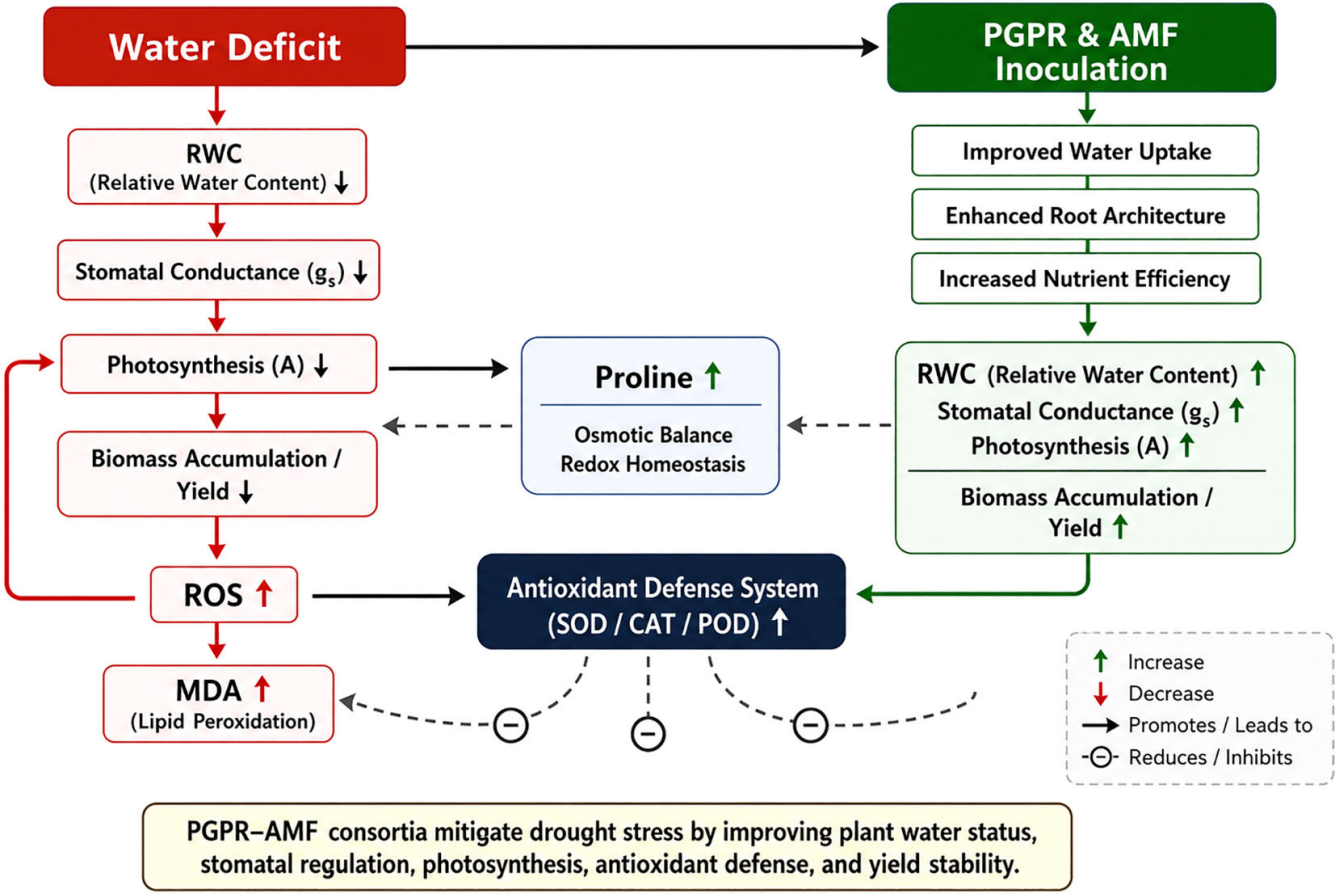
Conceptual mechanistic model summarizing the effects of water deficit and PGPR and AMF inoculation on plant water, physiological, and redox homeostasis, integrating changes in relative water content, stomatal conductance, photosynthesis, biomass accumulation and yield, as well as the modulation of the antioxidant system and oxidative damage. Created by the authors; the element is original.

However, fewer studies in maize have compared non-inoculated plants, single inoculations, and PGPR–AMF co-inoculation under graded water deficit while simultaneously integrating physiological, biochemical, growth, and yield-related traits. This limits a more mechanistic interpretation of how microbial consortia reorganize drought responses across levels of plant function. Therefore, this study evaluated whether PGPR–AMF co-inoculation improves drought tolerance in maize under moderate and severe water deficit. We hypothesized that the consortium would promote a more stable drought-response phenotype than single inoculations or the non-inoculated control, characterized by improved maintenance of water status, gas exchange, antioxidant regulation, and productivity. Based on these mechanisms, **Figure 1** summarizes the conceptual framework of the expected interactions between water deficit, PGPR–AMF inoculation, and the physiological, biochemical, and productive responses of maize plants.

## 2 Research methodology

### 2.1 Experimental site and plant material

The experiment was conducted under controlled greenhouse conditions in Quevedo, Los Ríos Province, Ecuador (approximately 1°02′S, 79°27′W). The study area is characterized by a tropical rainforest climate (Af) according to the Köppen–Geiger classification, with persistently warm temperatures and high atmospheric humidity typical of the Ecuadorian coastal lowlands (Peel et al., 2007).

Maize (*Zea mays* L.*)* seedlings were established under greenhouse management using uniform, healthy seeds. After emergence, seedlings with homogeneous vigor were selected to minimize initial variability among experimental units. Plants were grown individually in standardized plastic pots containing a homogenized substrate, maintaining identical container type and substrate volume across all experimental units. The substrate was homogenized before sowing to ensure comparable water-holding conditions among experimental units. Field capacity was determined gravimetrically for this specific substrate prior to water-deficit imposition; therefore, the moderate and severe deficit levels were defined relative to the water-holding capacity of the experimental substrate. One plant per pot was maintained throughout the experiment; therefore, each pot represented one experimental unit.

### 2.2 Experimental design and treatments

The experiment followed a completely randomized design (CRD) with a 4 × 3 factorial arrangement, allowing evaluation of the main effects of microbial inoculation and water availability, as well as their interaction, on physiological, biochemical, growth, and yield-related variables.

Factor A corresponded to microbial treatments: T0 (non-inoculated control), T1 (PGPR), T2 (AMF), and T3 (PGPR + AMF consortium). Factor B corresponded to water availability levels defined according to field capacity: E0 (100% field capacity), E1 (50–60% field capacity), and E2 (30–40% field capacity). The combination of both factors resulted in a total of 12 experimental treatments, which were randomly assigned to experimental units under controlled greenhouse conditions.

Each treatment combination included three biological replicates, resulting in a total of 36 experimental units. Each biological replicate corresponded to an independent plant grown in a separate pot, which was considered the experimental unit. Technical or subsampling measurements were performed when applicable to reduce within-unit measurement variability. For example, SPAD values were calculated as the mean of three readings per leaf, while gas-exchange measurements were recorded under standardized chamber conditions after signal stabilization. Therefore, statistical inference was based on biological replication, whereas repeated technical measurements were used to improve the precision of trait estimation within each experimental unit.

The resulting treatment combinations derived from the factorial arrangement are presented in **Table 1**, where each treatment code integrates the microbial inoculation level (T_0_–T_3_) and the corresponding water availability level (E_0_–E_2_). This structure represents all possible interactions between both factors and facilitates consistent comparison of physiological, biochemical, growth, and yield responses among treatments.

**Table 1:** Treatment combinations derived from the 4 × 3 factorial design.

Treatment code	Microbial treatment	Water regime
T0E0	Non-inoculated control	Optimal irrigation (100% field capacity)
T0E1	Non-inoculated control	Moderate water deficit (50–60 %)
T0E2	Non-inoculated control	Severe water deficit (30–40 %)
T1E0	PGPR	Optimal irrigation (100% field capacity)
T1E1	PGPR	Moderate water deficit (50–60%)
T1E2	PGPR	Severe water deficit (30–40%)
T2E0	AMF	Optimal irrigation (100% field capacity)
T2E1	AMF	Moderate water deficit (50–60%)
T2E2	AMF	Severe water deficit (30–40%)
T3E0	PGPR + AMF	Optimal irrigation (100% field capacity)
T3E1	PGPR + AMF	Moderate water deficit (50–60%)
T3E2	PGPR + AMF	Severe water deficit (30–40%)

The experiment was structured under a complete 4 × 3 factorial design, which included four levels of microbial inoculation (non-inoculated control, PGPR, AMF, and the PGPR + AMF consortium) and three levels of water stress (optimal irrigation, moderate water deficit, and severe water deficit). The combination of both factors resulted in a total of 12 experimental treatments, allowing an integrated evaluation of the individual and combined effects of microbial inoculation and water availability on the physiological and productive variables of the crop.

### 2.3 Microbial inocula and inoculation procedure

The microbial factor consisted of a plant growth-promoting rhizobacterium (PGPR) and an arbuscular mycorrhizal fungus (AMF), applied individually or in combination according to the experimental design (**Tables 1** and **2**). The PGPR treatment (T1) corresponded to *Bacillus subtilis*, whereas the AMF treatment (T2) corresponded to *Rhizophagus irregularis* (Ramírez-Flores et al., 2019; Bueno et al., 2022). The consortium treatment (T3) consisted of the combined application of both microorganisms, while T0 represented the non-inoculated control.

**Table 2:** Experimental factors and treatment levels (4 × 3 factorial design).

Factor	Code	Level / treatment	Description
Microbial treatment (A)	T_0_	Control	Plants without microbial inoculation
	T_1_	PGPR	Inoculation with plant growth-promoting rhizobacteria
	T_2_	AMF	Inoculation with arbuscular mycorrhizal fungi
	T_3_	PGPR + AMF	Combined inoculation (microbial consortium)
Water stress (B)	E_0_	Optimal irrigation	100 % of field capacity
	E_1_	Moderate deficit	50–60 % of field capacity
	E_2_	Severe deficit	30–40 % of field capacity

The table summarizes the factors and levels included in the 4 × 3 factorial CRD. Factor A corresponds to microbial inoculation (T_0_: non-inoculated control; T_1_: PGPR; T_2_: AMF; T_3_: PGPR + AMF consortium), and factor B corresponds to water availability levels defined as percentages of field capacity (E_0_: 100%; E_1_: 50–60%; E_2_: 30–40%). The factorial combination of both factors generated 12 treatments, enabling assessment of main effects and microbe × water-stress interactions across the physiological, biochemical, and productive variables.

The PGPR inoculum was applied as a liquid suspension at an approximate concentration of 1 × 10^8^ CFU mL^-1^ and delivered to the rhizosphere by soil drench at the base of each plant during the early vegetative stage, 7–10 days after emergence. The AMF inoculum was applied at approximately 200–300 spores per plant and incorporated directly into the substrate at the time of sowing to ensure early root colonization.

In the consortium treatment (T3), both inocula were applied using the same doses, timing, and procedures described for the individual treatments. All inoculation procedures were performed under controlled greenhouse conditions to ensure uniform establishment across experimental units.

To improve reproducibility, the source of the inocula was specified as a laboratory microbial collection for *Bacillus subtilis* and greenhouse trap cultures for *Rhizophagus irregularis*.

### 2.4 Water deficit imposition and sampling stage

Treatments were imposed after uniform seedling establishment at the early vegetative stage (approximately V3–V4)- Soil moisture treatments and field capacity determination were performed following the gravimetric approach described by Turner (1981). Pots were saturated and allowed to drain until drainage ceased to determine 100% field capacity, and irrigation was subsequently adjusted through periodic weighing to maintain the target moisture level for each treatment.

Water deficit was imposed gradually and maintained for 14 days before sampling. Plants assigned to E0 were maintained at 100% field capacity, those assigned to E1 at 50–60% field capacity, and those assigned to E2 at 30–40% field capacity throughout the defined stress period. This approach ensured stable separation among irrigation regimes and allowed the evaluation of maize physiological and biochemical responses under contrasting levels of water availability.

### 2.5 Trait assessment

#### 2.5.1 Physiological measurements

Relative chlorophyll content (SPAD) was estimated using a chlorophyll meter (SPAD-502Plus, Konica Minolta, Japan). SPAD readings were considered a non-destructive proxy for chlorophyll status rather than a direct quantification of chlorophyll concentration, since SPAD values may also be influenced by leaf thickness, leaf developmental stage, and nitrogen status. Readings were taken on the middle portion of the most recently fully expanded leaf, avoiding the midrib. For each experimental unit, SPAD was calculated as the mean of three readings taken from one leaf per plant to minimize within-leaf variability.

Net photosynthetic rate (A), stomatal conductance (gs), and transpiration rate (E) were measured using a portable infra-red gas analyzer (IRGA) (LI-6400XT Portable Photosynthesis System, LI-COR Biosciences, Lincoln, NE, USA) equipped with a standard leaf chamber. Measurements were performed on fully expanded leaves between 08:00 and 11:00 h to reduce diurnal effects. Chamber conditions were standardized across treatments at a photosynthetic photon flux density of 1000–1500 µmol m^-2^ s^-1^, reference CO_2_ concentration of 400 µmol mol^-1^, leaf temperature of 25 °C, and flow rate of 500 µmol s^-1^. The IRGA was zeroed and calibrated according to the manufacturer’s recommendations prior to each measurement session. A, gs, and E were recorded after signal stabilization, and values were expressed as µmol CO_2_ m^-2^ s^-1^, mol H_2_O m^-2^ s^-1^, and mmol H_2_O m^-2^ s^-1^, respectively.

Instantaneous water-use efficiency (WUE) was calculated as the ratio between net photosynthetic rate and transpiration rate (A/E). Relative water content (RWC) was determined according to the relative turgidity approach (Barrs and Weatherley, 1962), based on fresh weight, turgid weight, and dry weight of leaf tissue, and expressed as percentage.

#### 2.5.2 Biochemical assays

Redox homeostasis was characterized by quantifying the activity of key antioxidant enzymes, including superoxide dismutase (SOD), catalase (CAT), and peroxidases (POD), using leaf enzymatic extracts and standard spectrophotometric methods (Beauchamp and Fridovich, 1971; Aebi, 1984; Chance and Maehly, 1955). Oxidative damage was estimated by determining malondialdehyde (MDA) content as an indicator of lipid peroxidation (Heath and Packer, 1968; Hodges et al., 1999). Osmotic adjustment was assessed through the quantification of free proline using the acid-ninhydrin method (Bates et al., 1973).

For all biochemical determinations, leaf samples were collected from each experimental unit, immediately frozen in liquid nitrogen, and stored at −80 °C until analysis. All extraction steps were carried out on ice to limit enzymatic degradation and artefactual oxidation.

For enzyme extraction, approximately 0.10–0.20 g of frozen leaf tissue was ground to a fine powder in liquid nitrogen and homogenized in 1.0–2.0 mL of cold extraction buffer, typically consisting of 50 mM potassium phosphate buffer (pH 7.0–7.8), 1 mM EDTA, 1% (w/v) PVP, and 0.1% Triton X-100. Homogenates were centrifuged at 12,000–15,000 × g for 15–20 min at 4 °C, and the supernatant was used for enzyme assays. Soluble protein concentration was determined using Bradford assay with bovine serum albumin as standard when enzyme activity was expressed on a protein basis.

SOD activity was determined by the inhibition of nitro blue tetrazolium photoreduction using a total reaction volume of 3.0 mL, following standard spectrophotometric procedures. One unit of SOD activity was defined as the amount of enzyme required to cause 50% inhibition of NBT reduction under the assay conditions. Results were expressed as U mg^-1^ protein. CAT activity was assayed by monitoring the decomposition of H_2_O_2_ at 240 nm and expressed as U mg^-1^ protein. POD activity was quantified using the guaiacol oxidation method at 470 nm and expressed on a protein basis (Bates et al., 1973).

Lipid peroxidation was estimated as MDA concentration using the thiobarbituric acid reactive substances (TBARS) assay. Leaf tissue was homogenized in trichloroacetic acid, reacted with thiobarbituric acid, heated, cooled, and clarified by centrifugation. Absorbance was measured at 532 nm and corrected for non-specific turbidity at 600 nm. MDA concentration was calculated using the appropriate extinction coefficient and expressed as µmol g^-1^ FW.

Free proline was quantified spectrophotometrically following extraction in 3% sulfosalicylic acid, reaction with acid ninhydrin and glacial acetic acid, incubation at 100 °C for 60 min, and extraction of the chromophore into toluene. Absorbance was read at 520 nm, and proline concentration was reported as µmol g^-1^ fresh weight (FW).

#### 2.5.3 Growth measurements

Plant height was measured from the stem base to the uppermost point of the plant using a fixed criterion across treatments. At harvest, plants were separated into shoot and root fractions. Roots were gently washed to remove substrate, and both fractions were oven-dried at 65–70 °C to constant mass to obtain shoot and root dry biomass. Biomass allocation was summarised as the root:shoot ratio (R:S) calculated as root dry mass divided by shoot dry mass.

#### 2.5.4 Yield metrics

Yield was quantified at the end of the experimental period as grain yield per experimental unit, using a consistent harvest criterion across treatments. Harvest index (HI) was calculated as the ratio of yield metrics to total above-ground biomass (or total biomass, according to the study’s operational definition), ensuring the same denominator was applied to all treatments.

### 2.6 Data analysis

Prior to statistical analysis, all physiological, biochemical, growth, and yield-related variables were examined for consistency, missing values, and potential outliers. Data structure was inspected visually and analytically in order to verify the integrity of the dataset before inferential and multivariate procedures. Because the evaluated traits were measured in different units and scales, variables were standardized using a z-score transformation, in which each observation was centered by the mean of its variable and divided by the corresponding standard deviation. This procedure ensured comparability across variables and prevented those with larger numerical magnitudes from exerting disproportionate influence on the multivariate analyses.

Given the factorial structure of the experiment, treatment effects were first assessed using two-way analysis of variance (ANOVA), considering microbial inoculation treatment, water regime, and their interaction as fixed factors. This inferential step was included to evaluate the individual and combined effects of both experimental factors on the measured response variables before proceeding to integrative multivariate analysis. When significant differences were detected, treatment means were separated using Tukey’s honestly significant difference (HSD) test at a significance threshold of α = 0.05. This approach allowed robust comparison among treatment combinations while controlling the family-wise error rate (Mircea et al., 2023).

To further explore trait associations under the different treatment conditions, Spearman’s rank correlation coefficient (ρ) was calculated among physiological, biochemical, growth, and yield-related variables. This non-parametric approach was selected because several variables did not simultaneously satisfy normality assumptions and because it is appropriate for identifying monotonic relationships without requiring strict linearity. Correlation analysis provided an initial overview of the functional relationships among variables linked to plant water status, antioxidant metabolism, osmotic adjustment, biomass allocation, and productivity.

To summarize the multivariate structure of the dataset and identify the main gradients of biological variation, principal component analysis (PCA) was performed using the correlation matrix of the standardized variables (Mircea et al., 2023). PCA was used as a dimensionality reduction technique to condense the information contained in the original variables into orthogonal components that maximized explained variance. The selection of relevant components was based on their individual contribution and cumulative explained variance, prioritizing components that together accounted for a substantial proportion of total variability and that retained meaningful biological interpretation.

For graphical interpretation of treatment distribution and variable contribution, the first two principal dimensions were represented through an HJ-Biplot, which allows simultaneous visualization of treatments and variables in the same reduced multivariate space (Yan and Tinker, 2006). This representation was used to identify functional groupings, treatment separation patterns, and the relative contribution of physiological and biochemical traits to the observed response structure. In addition, heatmaps combined with hierarchical cluster analysis were used as complementary exploratory tools to identify response modules and groups of variables with similar behavior across treatments. This approach facilitated recognition of coordinated physiological patterns and supported the biological interpretation of the correlation and PCA outputs.

The overall statistical workflow, including preprocessing, inferential analysis, and multivariate integration, is summarized in **Table 3**.

**Table 3:** Statistical analysis workflow and inference criteria.

Stage	Method	Justification / theoretical support
Data preprocessing	Outlier inspection, distribution assessment, standardization (z-score)	Ensures comparability among variables with different units and prevents high-magnitude variables from dominating multivariate analyses.
Treatment effects	Two-way ANOVA + Tukey’s HSD	Tests the main effects of inoculation, water regime, and their interaction before multivariate integration.
Correlation analysis	Spearman’s correlation (ρ)	Appropriate for non-normal data; allows identification of monotonic relationships among physiological, biochemical, and productive variables.
Dimensionality reduction	Principal Component Analysis (PCA) based on the correlation matrix	Synthesizes multivariate variability and reveals the main functional gradients of the system.
Component selection	Explained variance and cumulative variance (>70%)	Ensures adequate representation of total variability with biologically meaningful interpretation.
Multivariate visualization	HJ-Biplot (Dim1 × Dim2)	Enables simultaneous interpretation of variables and treatments; vector geometry reveals functional associations.
Pattern discovery	Heatmaps and hierarchical cluster analysis	Identifies response modules and groups of variables with similar physiological behavior.
Results presentation	Correlation coefficients, explained variance, multivariate projections	Promotes an integrated biological interpretation beyond univariate tests.

**Table 3** summarizes the statistical workflow established *a priori* for the analysis of physiological, biochemical, growth, and yield-related variables. Inferential outputs, including ANOVA, *post hoc* comparisons, correlation coefficients, explained variance of principal components, multivariate projections, and clustering patterns, are presented in the Results and Discussion sections. This workflow was defined to ensure methodological consistency, analytical reproducibility, and biologically integrated interpretation of plant responses to water deficit and microbial inoculation.

All statistical analyses were conducted in R using the packages readxl, dplyr, FactoMineR, factoextra, corrplot, Hmisc, ggplot2, pheatmap, and the base stats package. Statistical significance was considered at p ≤ 0.05.

This analytical framework was defined *a priori* to ensure methodological consistency and reproducibility across all response variables evaluated in the study.

## 3 Results

Two-way ANOVA showed significant effects of microbial treatment and water regime on yield, net photosynthetic rate, relative water content (RWC), and SOD activity. Yield was affected by treatment and water regime (F = 18.27 and 46.20, respectively; p < 0.001 in both cases). Net photosynthetic rate was also affected by both factors (F = 6.99, p = 0.0015; F = 26.81, p < 0.001), as were RWC (F = 15.97, p < 0.001; F = 7.43, p = 0.0031) and SOD activity (F = 6.66, p = 0.0020; F = 18.09, p < 0.001). In contrast, proline and MDA were mainly affected by stress intensity (F = 54.39 and 109.83, respectively; p < 0.001), whereas treatment × water-regime interactions were weak overall, with MDA approaching significance (F = 2.47, p = 0.0526). Under severe water deficit (E2), Tukey’s HSD showed higher yield in T3 than in T0 (mean difference = 1.42; p = 0.0466).

**Figure 2:**
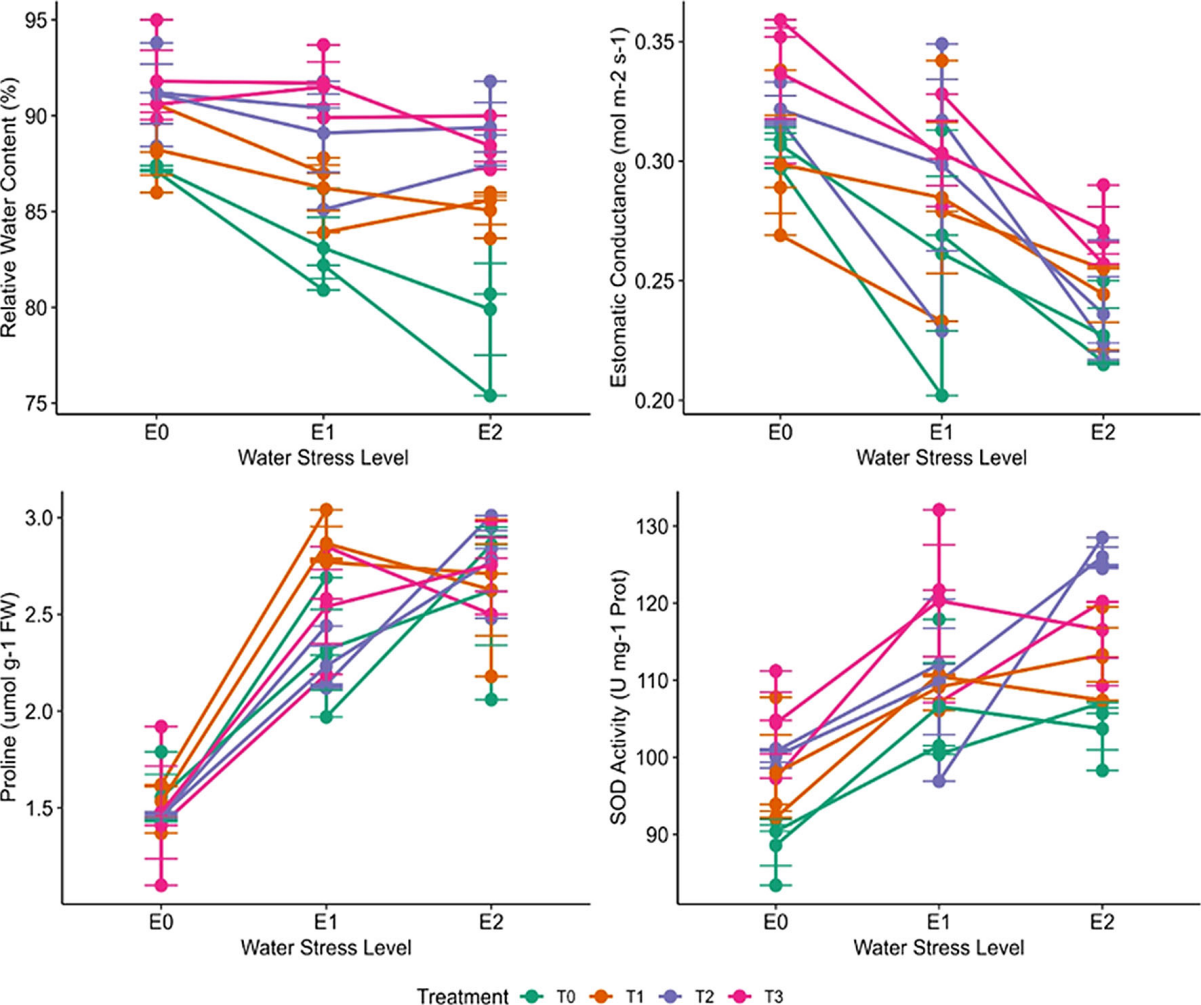
Physiological and biochemical responses of plants to different levels of water stress under distinct microbial treatments.

Across treatments, progressive water restriction reduced chlorophyll status, gas exchange, plant water status, and final productivity (**Figure 2**). Under severe stress, T3 maintained a mean RWC of 88.4%, compared with 79.9% in T0. At the same stress level, T3 also showed higher photosynthetic rate (23.76 vs. 21.71 µmol CO_2_ m^-2^ s^-1^), higher stomatal conductance (0.271 vs. 0.227 mol H_2_O m^-2^ s^-1^), and higher yield (8.71 vs. 7.29) t ha^-1^. Tukey comparisons indicated higher RWC in T2 and T3 than in T0 (p = 0.0079 and p = 0.0145, respectively), whereas yield was significantly higher only in T3 than in T0 (p = 0.0466).

Biochemical responses intensified with water deficit, although their magnitude differed among microbial treatments (**Figure 2**). SOD activity increased from 88.6 U mg^-1^ protein in T0E0 to 103.7 U mg^-1^ protein in T0E2 and reached 116.5 U mg^-1^ protein in T3E2. The highest SOD value under severe stress was recorded in T2E2 (126.0 U mg^-1^ protein). Proline increased progressively with stress across all treatments, reaching 2.62 µmol g^-1^ FW in T0E2 and 2.76 µmol g^-1^ FW in T3E2. MDA values under severe stress were 4.74 in T0E2 and 4.51 in T3E2.

**Figure 3:**
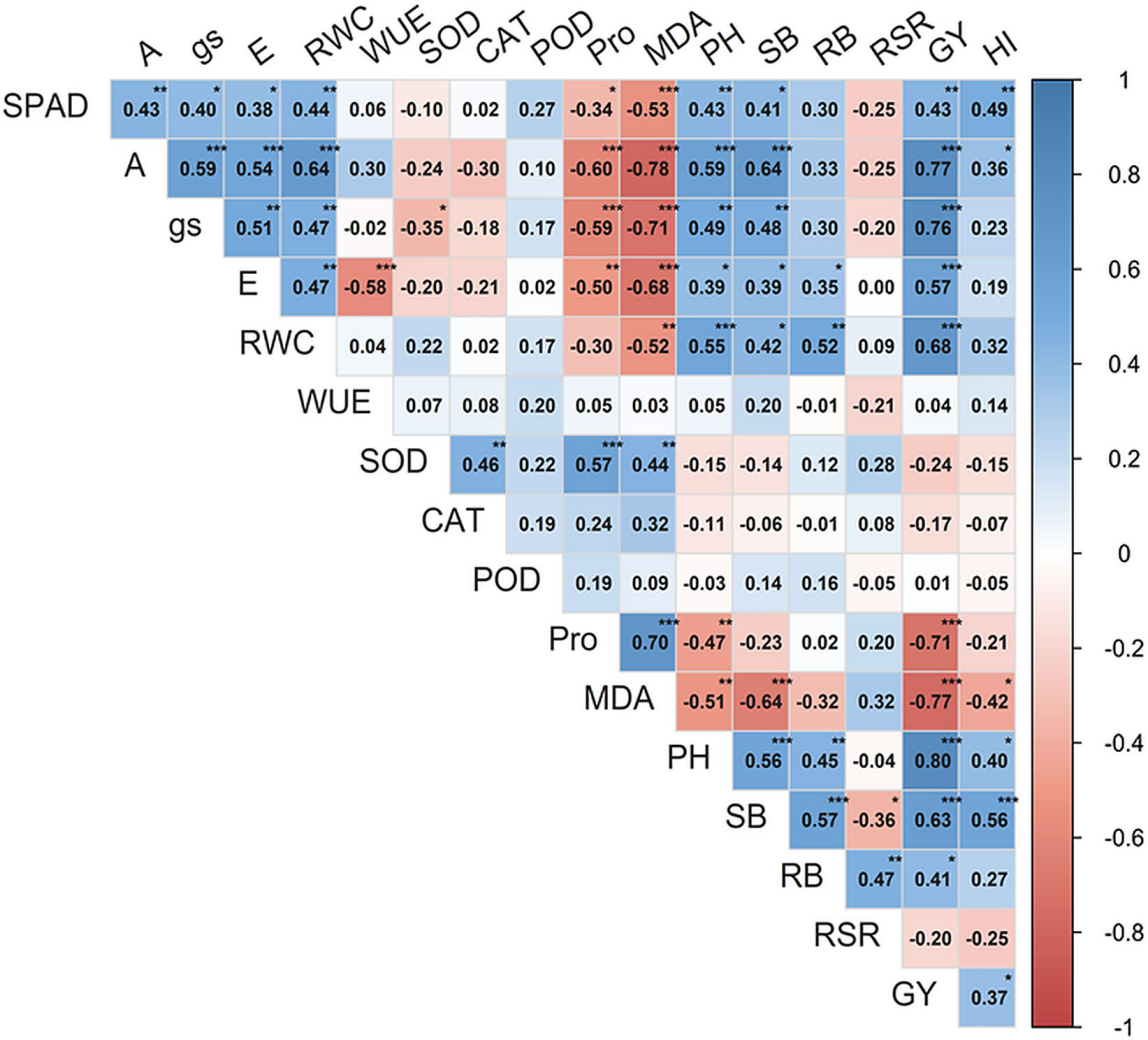
Spearman correlation analysis highlighting proline-associated homeostasis.

The Spearman correlation matrix showed a compact block of positive and highly significant correlations (ρ > 0.6; p < 0.01) among SPAD, photosynthesis, yield, shoot biomass, root biomass, plant height, and harvest index (**Figure 3**). In contrast, proline and MDA showed negative correlations with yield, photosynthesis, RWC, and growth-related traits. Antioxidant enzymes (SOD, CAT, and POD) were positively associated with proline and MDA. Stomatal conductance, transpiration, and water-use efficiency occupied intermediate positions within the correlation structure.

### 3.1 Variable importance and multivariate structure

**Figure 4:**
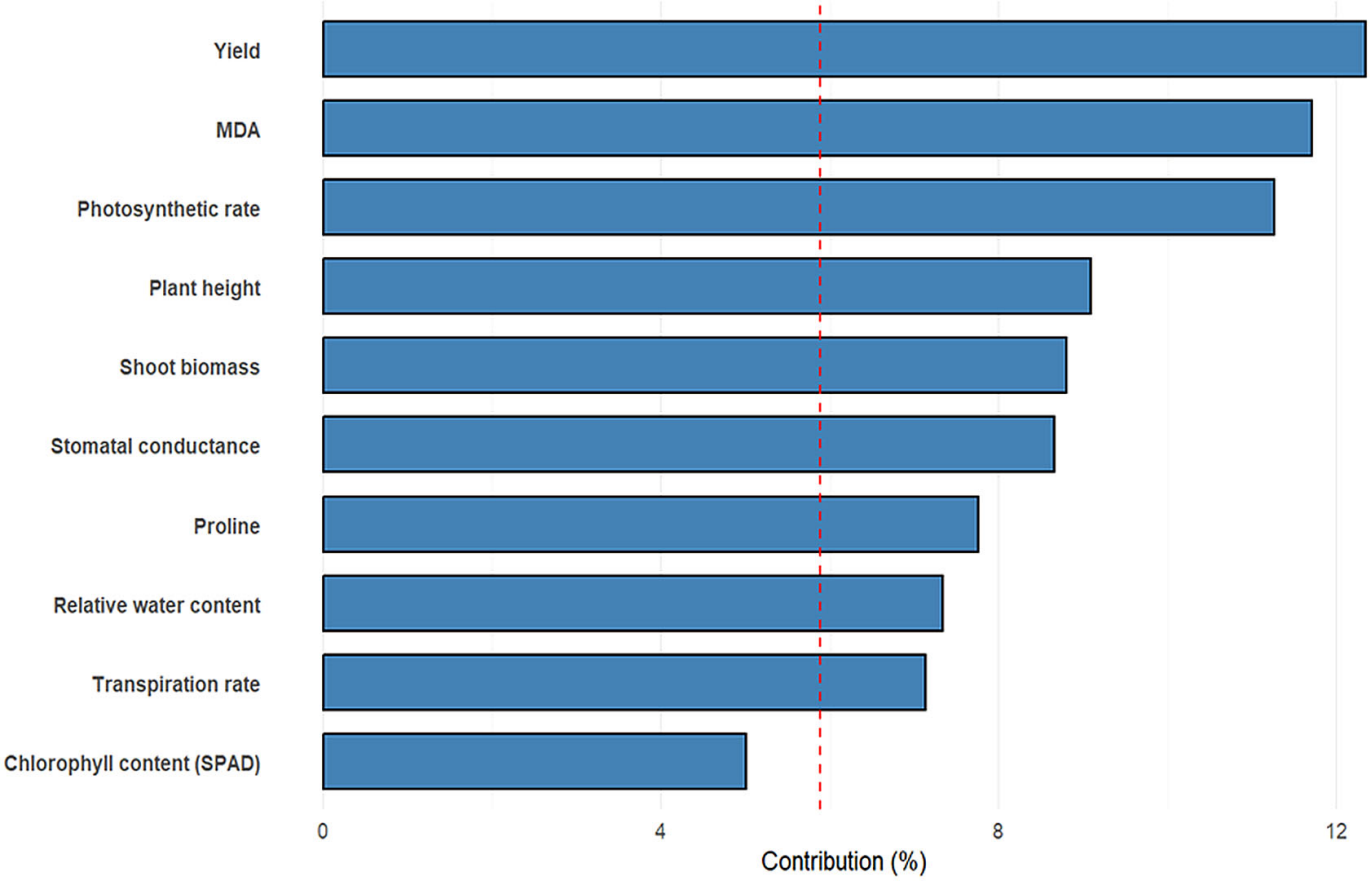
Quantification of variable importance in response to water deficit.

The variable-importance analysis identified yield (12.3%), MDA (11.8%), and net photosynthetic rate (11.2%) as the variables with the highest relative contribution to maize responses under water deficit (**Figure 4**). Plant height (9.1%) and shoot biomass (8.8%) also contributed substantially. Stomatal conductance, proline, RWC, and transpiration showed intermediate contributions, whereas SPAD had the lowest relative contribution (~5.1%).

**Figure 5:**
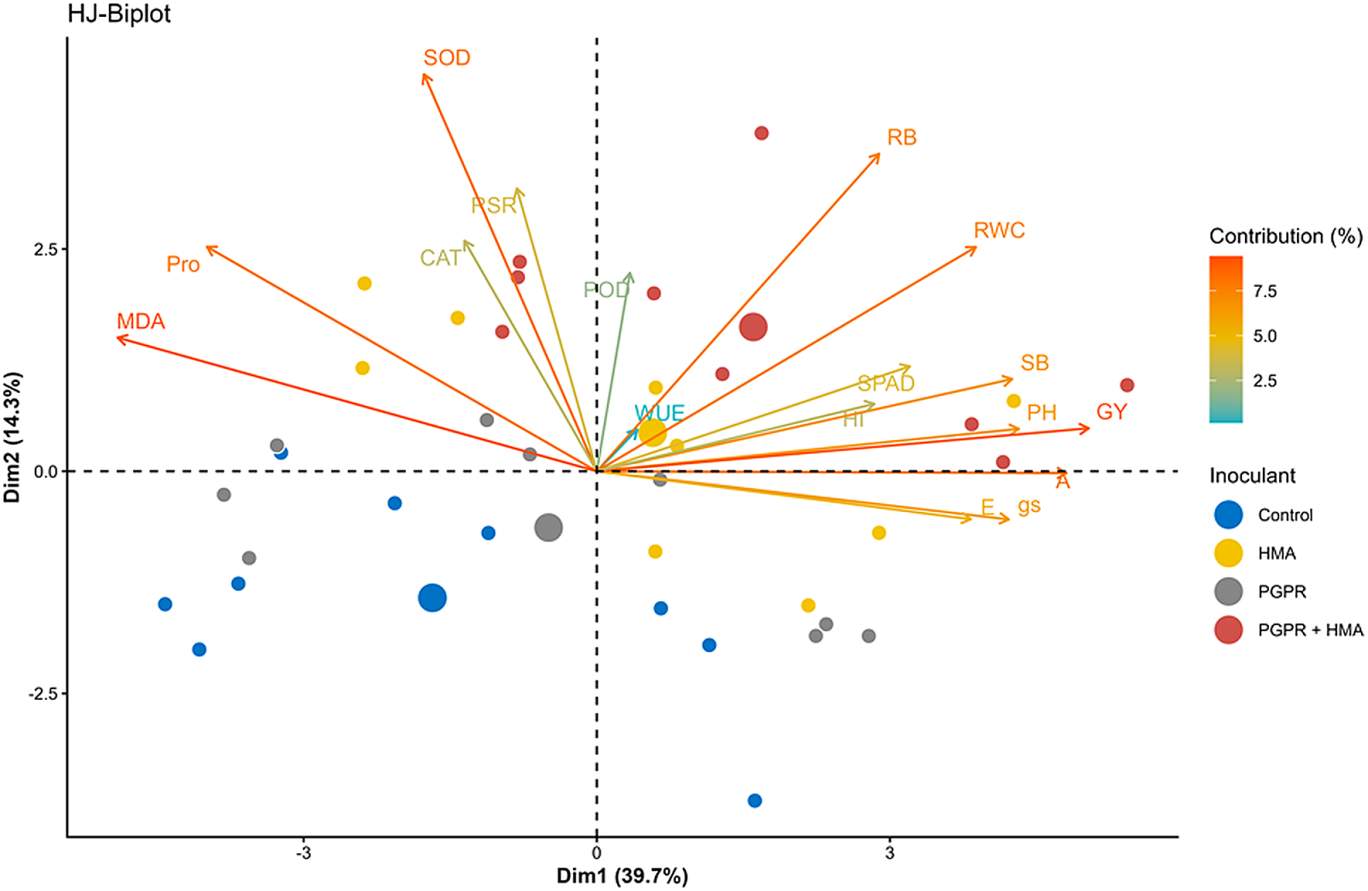
HJ-biplot analysis of redox homeostasis and water status. The abbreviations used in the HJ-Biplot correspond to physiological, biochemical, and productive variables associated with plant water status and redox balance: SPAD, relative chlorophyll content; A, net photosynthetic rate; gs, stomatal conductance; E, transpiration rate; RWC, relative water content; WUE, water-use efficiency; SOD, superoxide dismutase; CAT, catalase; POD, peroxidases; Pro, proline; MDA, malondialdehyde; PH, plant height; SB, shoot biomass; RB, root biomass; RSR, root-to-shoot ratio; GY, grain yield; and HI, harvest index.

PCA reduced the 17 evaluated variables to two main axes that explained 54.0% of the total variance (Dim1 = 39.7%, Dim2 = 14.3%) (**Figure 5**). Dim1 separated productivity-related variables, including photosynthesis, stomatal conductance, transpiration, SPAD, shoot biomass, RWC, and yield, from oxidative and osmotic stress markers, mainly MDA and proline. Dim2 was defined mainly by SOD, CAT, POD, and root-to-shoot ratio.

Although the first two dimensions captured the dominant physiological and oxidative gradients of the system, the inclusion of Dim3 increased the cumulative explained variance to 74.0% (**Table 4**) and provided additional biological insight related to structural adjustment and resource-use strategies under drought. The main contributors to Dim3 were root biomass (18.4%), root-to-shoot ratio (15.9%), WUE (13.7%), CAT (11.2%), and POD (9.8%), indicating that this axis captured complementary mechanisms associated with biomass allocation, water-use optimization, and antioxidant regulation. The first three dimensions together explained 74.0% of cumulative variance (**Table 5**).

**Table 4:** Variance explained by the PCA dimensions.

Dimension	Explained variance (%)	Cumulative variance (%)	General interpretation
Dim1	39.7	39.7	Gradient of physiological and productive efficiency
Dim2	14.3	54.0	Antioxidant regulation and biochemical response
Dim3	20.0	74.0	Structural adjustments and biomass allocation

The inclusion of the third dimension allowed the commonly accepted threshold of cumulative explained variance (>70%) to be exceeded, thereby strengthening the ability of the analysis to capture the complexity of the plant–microorganism system under water deficit conditions.

**Table 5:** Variables with the highest contribution to PCA dimension 3.

Variable	Abbreviation	Contribution (%)	Functional interpretation
Root biomass	RB	18.4	Increased soil exploration
Root-to-shoot ratio	RSR	15.9	Biomass redistribution under stress
Water use efficiency	WUE	13.7	Physiological optimization of water consumption
Catalase	CAT	11.2	Complementary antioxidant regulation
Peroxidases	POD	9.8	Protection against oxidative stress

These variables did not respond directly to the main water-stress gradient but rather reflected secondary structural and physiological mechanisms that complement the immediate responses captured by the first two dimensions.

### 3.2 Redox homeostasis patterns

**Figure 6:**
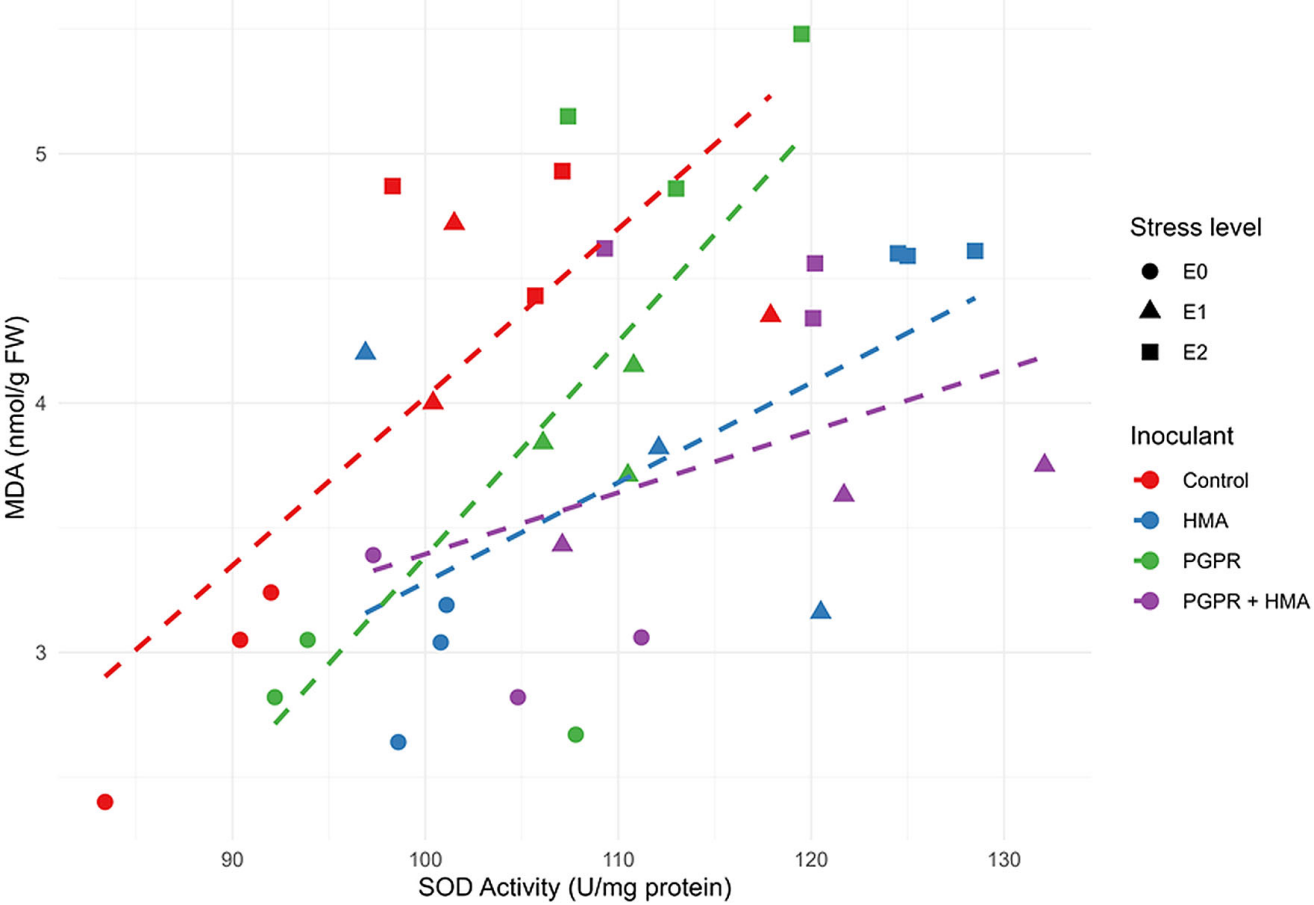
Characterization of redox homeostasis: Relationship between SOD activity (defense) and MDA accumulation (damage).

The relationship between SOD activity and MDA content differed among microbial treatments (**Figure 6**). In the control treatment, increasing SOD activity was accompanied by increasing MDA. In inoculated treatments, particularly T3, lower MDA values were observed at comparable SOD levels. Under severe water deficit, T3 combined high SOD activity with lower MDA than T0.

**Figure 7:**
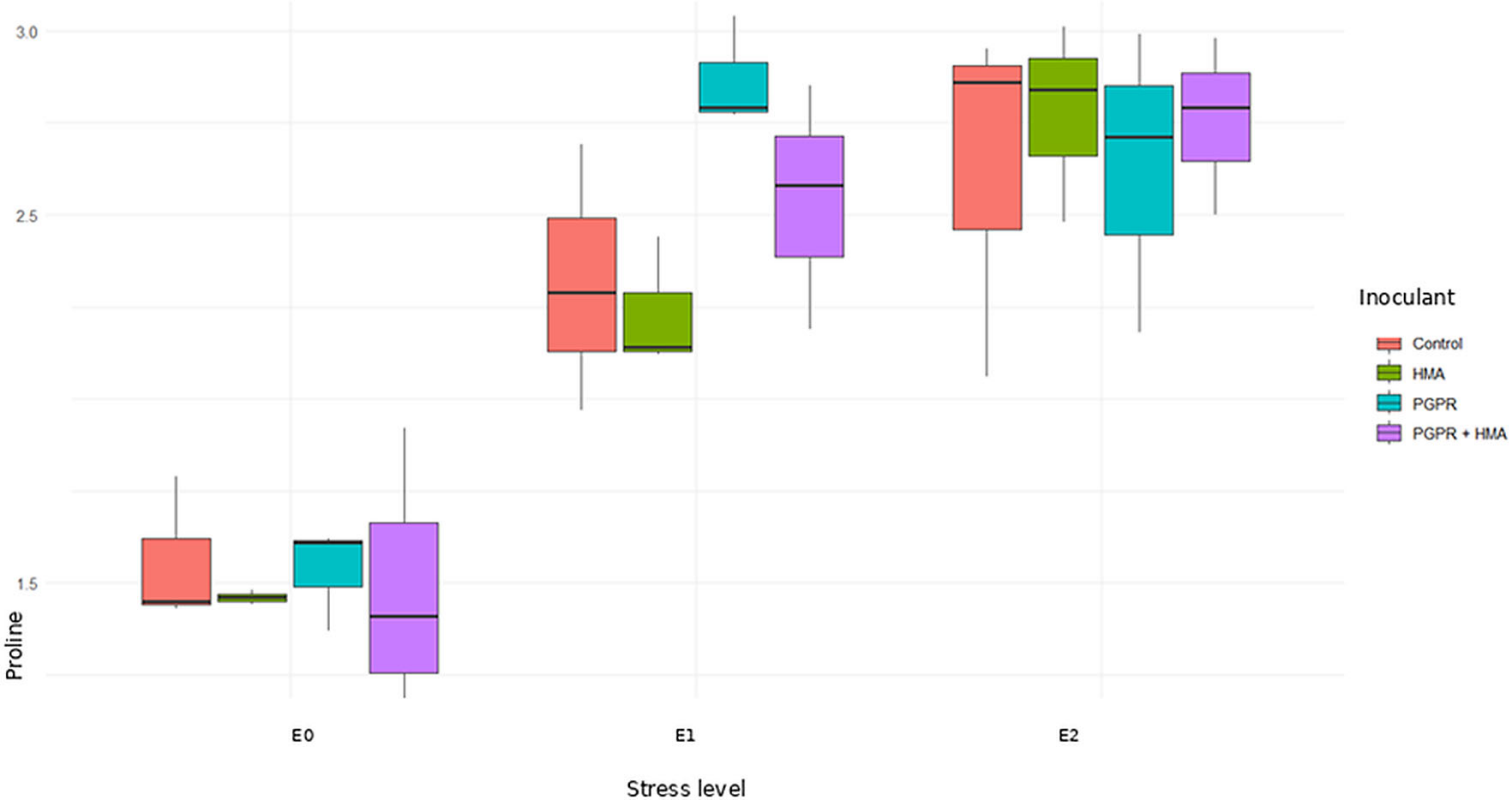
Proline accumulation across treatments and stress levels.

Proline increased consistently with drought intensity across all microbial treatments (**Figure 7**). Under severe water deficit, the highest mean values were recorded in T2 and T3 (2.78 and 2.76 µmol g^-1^ FW, respectively), whereas T0 reached 2.62 µmol g^-1^ FW.

Contribution of variables to Dimension 3.

Since Dimension 3 was not graphically represented together with the first two dimensions, its interpretation was addressed through a contribution table, allowing the identification of the variables that defined this additional axis. This dimension was dominated by variables related to root architecture, biomass allocation, and resource-use efficiency, reflecting medium-term adaptive strategies.

Proline accumulation increased consistently with drought intensity across all microbial treatments (**Figure 7**). Under severe water deficit, the highest mean values were observed in T2 and T3 (2.78 and 2.76 µmol g^-1^ FW, respectively), whereas the non-inoculated control reached 2.62 µmol g^-1^ FW.

## 4 Discussion

The central finding of this study is that microbial inoculation, particularly the PGPR–AMF consortium, improved drought resilience in maize by preserving plant water status, gas exchange, and yield under water-limited conditions. Although drought intensity remained the dominant factor driving the accumulation of stress markers such as proline and MDA, inoculated plants maintained a more favorable physiological balance than the non-inoculated control, especially under severe stress. This distinction is important because it indicates that the beneficial effect of inoculation was not limited to one isolated trait, but involved coordinated responses across physiological, biochemical, and productive levels. In our dataset, the consortium treatment maintained higher relative water content, higher photosynthetic activity, and higher yield under severe water deficit, together with lower oxidative damage than the control. This result supports the increasingly consolidated view that effective drought mitigation does not depend on the exacerbated activation of a single defense pathway, but rather on the coordinated integration of multiple physiological and biochemical functions (Ahmad et al., 2022; Chieb and Gachomo, 2023; Notununu et al., 2024; Aamir et al., 2026).

The superiority of the consortium over the non-inoculated treatment can be interpreted in terms of functional complementarity between PGPR and AMF. PGPR are known to influence root architecture, nutrient mobilization, hormone balance, and stress signaling, while AMF improve water and nutrient acquisition through extraradical hyphae and contribute to the regulation of hydraulic and antioxidative processes (Vacheron et al., 2013; Van de Poel and Van Der Straeten, 2014; Chandrasekaran, 2022). When both partners are combined, the resulting phenotype may reflect a more integrated belowground–aboveground adjustment, where improved root functioning supports leaf-level physiological stability. This is consistent with recent evidence indicating that co-inoculation outperforms single inoculation in improving drought tolerance in maize and other crops by acting in concert on the plant’s water status, growth, nutrition, and oxidative balance (Ouhaddou et al., 2024; Savastano and Bais, 2024a; Shams et al., 2026). However, the present results also suggest that synergy should not be assumed *a priori*. The fact that the interaction effect was weak for several traits indicates that water deficit itself remained the primary source of variance, while microbial inoculation modulated the magnitude and expression of the stress response. This fits with recent literature emphasizing that outcomes of AMF–PGPR combinations depend on inoculum compatibility, host genotype, and environmental severity (Khan et al., 2024; Savastano and Bais, 2024a).

One of the most relevant patterns in this study was the maintenance of relative water content and photosynthetic performance in inoculated plants under severe drought. Under water deficit, stomatal closure limits transpiration and CO_2_ diffusion, which immediately reduces photosynthetic carbon assimilation and often initiates a cascade of metabolic constraints that affect biomass accumulation and yield (Fang and Xiong, 2015; Sheoran et al., 2022). In this context, the higher RWC observed in inoculated plants indicates that microbial treatments helped buffer dehydration at the tissue level. This may have contributed directly to the preservation of stomatal function and leaf gas exchange, thereby sustaining a larger fraction of the assimilatory capacity of the crop. AMF have repeatedly been associated with improved plant water status under drought through effects on root hydraulic conductivity, aquaporin regulation, and improved soil exploration, while PGPR can stimulate root growth and hormone-mediated adjustments that favor water uptake and stress (Ruiz-Lozano et al., 2009; Quiroga et al., 2017; Chandrasekaran, 2022). The positive association observed here among photosynthesis, stomatal conductance, transpiration, relative water content, biomass, and yield supports the interpretation that preservation of water relations was not merely a descriptive trait, but a central determinant of productive stability under drought.

This interpretation is strengthened by the position of physiological variables within the correlation matrix and the multivariate ordination. Traits associated with carbon gain and growth were clustered together and clearly opposed to oxidative and osmotic stress markers, indicating that the most favorable treatment combinations were those able to preserve an actively functioning physiological state rather than simply survive stress. Water-use efficiency occupied an intermediate position, suggesting that it contributed to drought acclimation, but not independently of the broader maintenance of photosynthetic performance. This is consistent with the notion that WUE should be interpreted cautiously, because higher WUE can arise either from productive assimilation or from a strong reduction in transpiration, and these two scenarios do not have the same agronomic meaning (Blum, 2009; Montilla-Bascón et al., 2017). In the present study, the positive association of WUE with productive variables suggests that inoculated plants were not merely restricting water loss, but were maintaining a more favorable balance between carbon assimilation and transpiration. Such coordination is particularly relevant in maize, where productive losses under drought are strongly linked to failures in maintaining functional gas exchange during critical growth periods (Usmani et al., 2020; Sheoran et al., 2022).

Another key contribution of this study lies in the interpretation of redox homeostasis. Drought stress frequently disrupts electron transport in chloroplasts and mitochondria, increases photorespiratory pressure, and favors the overproduction of reactive oxygen species. When ROS generation exceeds the detoxification capacity of the cell, oxidative damage accumulates, affecting membranes, proteins, and metabolic integrity (Ramachandra Reddy et al., 2004; Hasanuzzaman et al., 2020; Mittler et al., 2022). The increase in antioxidant activity observed here, particularly for SOD, CAT, and POD, is therefore consistent with a compensatory defense response to drought-induced oxidative pressure. However, antioxidant activation alone should not be interpreted as equivalent to tolerance. In our dataset, the biologically most informative pattern was not simply the increase in antioxidant enzymes, but the relationship between enzymatic defense and oxidative damage. Inoculated plants, and especially those receiving the consortium, combined relatively high antioxidant activity with lower MDA accumulation than the non-inoculated control under severe stress. This indicates a more effective control of oxidative stress, in which enzymatic activation was associated with reduced membrane lipid peroxidation rather than merely reflecting greater damage intensity (Laxa et al., 2019; Gowtham et al., 2022; Zou et al., 2021).

The distinction between antioxidant activation and effective redox regulation is important for interpreting the differences among microbial treatments. In our results, AMF alone reached the highest absolute SOD activity under severe drought, whereas the consortium showed the most favorable combination of water status, yield maintenance, and lower oxidative damage. This suggests that the single AMF treatment may have strongly stimulated the antioxidant machinery, but the consortium achieved a more integrated phenotype in which oxidative protection was coupled to superior physiological function. Such a pattern is compatible with the idea that drought tolerance is not determined by the amplitude of one enzyme response, but by the coordination among ROS production, detoxification, membrane stability, and maintenance of assimilation (Hasanuzzaman et al., 2020; Laxa et al., 2019). It also agrees with reports that microbial inoculation can alleviate drought not only by activating antioxidants, but also by reducing stress load through improved water uptake, nutrient acquisition, and stomatal regulation, thereby lowering the need for emergency detoxification responses (Azizi et al., 2022; Ouhaddou et al., 2024). From this perspective, the lower MDA values observed in consortium-inoculated plants at comparable SOD levels are particularly meaningful because they point to improved stress containment rather than simply stronger biochemical reactivity.

The accumulation of proline deserves a similarly nuanced interpretation. Proline is widely recognized as a multifunctional stress-associated metabolite involved in osmotic adjustment, ROS buffering, redox balance, and protection of proteins and membranes (Hayat et al., 2012; Szabados and Savouré, 2010). In the present study, proline increased progressively with drought intensity in all treatments, confirming that it was a sensitive marker of water deficit. However, its negative association with photosynthesis, relative water content, and yield indicates that higher proline mainly tracked stress severity rather than superior productive performance. This agrees with the broader literature showing that proline accumulation can be beneficial as part of the drought-response syndrome, but should not be interpreted in isolation as proof of agronomic tolerance (Blum, 2009; Ghosh et al., 2021). In practice, a drought-tolerant phenotype is better characterized by the simultaneous maintenance of water relations, metabolic activity, and yield than by the isolated elevation of osmolytes. The present results therefore support the view that proline played an adaptive but secondary role, whereas the principal determinants of productive stability were the preservation of photosynthesis and the mitigation of oxidative damage.

The multivariate analyses helped synthesize this hierarchy of responses and provided a more integrated interpretation than univariate analysis alone. The PCA and HJ-Biplot clearly separated productivity-related traits from oxidative and osmotic stress markers, with the consortium treatment projected toward the multivariate space associated with superior physiological functioning. This geometric arrangement reinforces the conclusion that drought tolerance in this system was organized along a dominant axis contrasting functional performance versus stress burden. Similar analytical approaches have been useful in drought studies because they allow the simultaneous interpretation of correlated traits and reduce the risk of overemphasizing isolated variables that may not reflect the whole-plant phenotype (Mohi-Ud-Din et al., 2021; Mircea et al., 2023). In the present case, the multivariate structure confirms that yield preservation under drought depended primarily on maintaining a coherent physiological state, characterized by higher photosynthetic capacity, better hydration, and lower oxidative injury, rather than on maximizing single defense markers. This is also why MDA emerged as a particularly informative variable: its opposition to yield-related traits indicates that membrane stability was tightly linked to performance under stress.

From an agronomic perspective, these findings support microbial inoculation, particularly the PGPR–AMF consortium, as a promising biological strategy for drought-prone maize production systems. The practical value of this response lies in the fact that the consortium improved not only biochemical indicators, but also yield-associated outcomes under severe water deficit. This is essential, because many stress-mitigation studies document biochemical shifts without demonstrating clear productive relevance. At the same time, the results obtained should be interpreted considering that the study was developed under controlled greenhouse conditions, so their extrapolation to field environments requires additional validation, given that soil, microbial and climatic variability could modify the magnitude and stability of the observed responses (Khan et al., 2026). It will also be important to report inoculum identity, dose, and colonization dynamics with full precision, because the success of microbial technologies depends strongly on formulation, compatibility, and environmental fit (Savastano and Bais, 2024a; Ouhaddou et al., 2024). Even with these limitations, the present results provide strong evidence that drought mitigation in maize is best understood as a coordinated physiological process in which microbial inoculation supports water status, gas exchange, redox balance, and, ultimately, yield stability.

## 5 Conclusion

In summary, factorial analysis showed that water regime was the dominant driver of proline and MDA accumulation, whereas both water regime and microbial treatment significantly shaped relative water content, photosynthesis, SOD activity, and yield. Under severe water deficit, the PGPR–AMF consortium maintained 10.7% higher relative water content and 19.5% higher yield than the non-inoculated control.

These findings indicate that drought mitigation in maize depends on coordinated maintenance of plant water status, gas exchange, and redox balance rather than on isolated antioxidant activation. From an agronomic perspective, combined inoculation was more resilient than the non-inoculated treatment under restricted irrigation and therefore represents a promising biological option for drought-prone production systems.

Future studies should validate these responses under field conditions and report strain-level inoculum identity, dose, and colonization dynamics to strengthen reproducibility and translational applicability.

## Data Availability

The raw data supporting the conclusions of this article will be made available by the authors, without undue reservation.
